# DLX3 (Q178R) mutation delays osteogenic differentiation via H19/miR-29c-3p/KDM5B axis in TDO-iPSCs-derived MSCs

**DOI:** 10.1016/j.gendis.2023.05.011

**Published:** 2023-07-03

**Authors:** Liying Dong, Na Zhao, Dongmei Wang, Meng Wang, Yixin Zhang, Liangjie Sun, Chong Ding, Yixiang Wang, Zeyun Ma

**Affiliations:** a Central Laboratory, Peking University School and Hospital of Stomatology, Beijing 100081, China; b Department of Oral and Maxillofacial Surgery, Peking University School and Hospital of Stomatology, Beijing 100081, China; c National Engineering Laboratory for Digital and Material Technology of Stomatology, Peking University School and Hospital of Stomatology, Beijing 100081, China; d Beijing Key Laboratory of Digital Stomatology, Peking University School and Hospital of Stomatology, Beijing 100081, China; e The Second Clinical Division, Peking University School and Hospital of Stomatology, Beijing 100081, China; f Department of VIP Service, Peking University School and Hospital of Stomatology, Beijing 100081, China; g Department of Restorative Dentistry and Biomaterials Sciences, Harvard School of Dental Medicine, Boston, MA 02115, USA; h Department of Prosthodontics, Fudan University, Shanghai 200001, China; i Shanghai Key Laboratory of Craniomaxillofacial Development and Diseases, Shanghai Stomatological Hospital, Fudan University, Shanghai 200001, China

Tricho-dento-osseous (TDO) syndrome is a rare autosomal dominant disease resulting from distal-less homeobox 3 (DLX3) mutation.^1^^,^^2^ Accumulative bone density in alveolar bone is a clinically favorable phenotype for TDO patients. However, the limited number of bone marrow mesenchymal stem cells (BMSCs) in TDO patients restricts their application. Since TDO-specific induced pluripotent stem cells (iPSCs) can yield a large variety of patient cells as the important cell source to investigate specific tissue/organ development, establish disease models, and develop new treatment approaches, we established TDO-iPS cells with mutant DLX3 (c.533A > G, Q178R) of the host cells. Our previous research indicated that mutant DLX3 could down-regulate H19 to regulate bone formation.^3^ In this study, we successfully generated and verified TDO-iPSCs, and compared the osteogenic differentiation capacity from TDO-iPSCs to mesenchymal stem cells (MSCs) and from normal human iPSCs to iPS-MSCs. We also predicted that H19 could sponge miR-29c-3p and lysine demethylase 5B (KDM5B) was the target gene of miR-29c-3p. We found transfected miR-29c-3p inhibitor down-regulated miR-29c-3p and up-regulated the expression of KDM5B and osteogenic biomarker. Previous research suggested that KDM5B promoted osteo-differentiation by binding to the promoter region of alkaline phosphatase (ALP), RUNX family transcription factor 2 (RUNX2), and osteocalcin (OCN).^4^ We deduced that H19, miR-29c-3p, and KDM5B, together serving as a ceRNA (competing endogenous RNA) network, were regulated by DLX3 to affect osteo-differentiation, which is a novel pathway to regulate bone formation and shed light on the treatment of bone-related diseases.

Firstly, we obtained alveolar bone tissue from a TDO patient by alveoloplasty. The tissue was used to isolate BMSCs and the second passage of BMSCs was subjected to iPS induction by transduction with a set of Sendai viral constructs, and DLX3 mutation identification through genomic DNA sequencing. Cells with an embryonic stem cell-like morphology were first visible at 15 days after transduction. The colonies were collected at 20 days after transduction and transferred into a 24-well plate coated with Matrigel in Reproeasy complete medium containing bFGF and Y27632. The reprogramming of TDO-BMSCs was shown in Figure 1A. Karyotype analysis showed that these colonies were 46, XX by G-banding. Genomic DNA sequencing showed that the colonies had the same DLX3 (Q178R) mutation as BMSCs from the TDO patient (Fig. S1), which indicated that the sub-cloned iPS cells were from the TDO patient. We used the TDO-iPS cells for further characterization.

**Figure 1:**
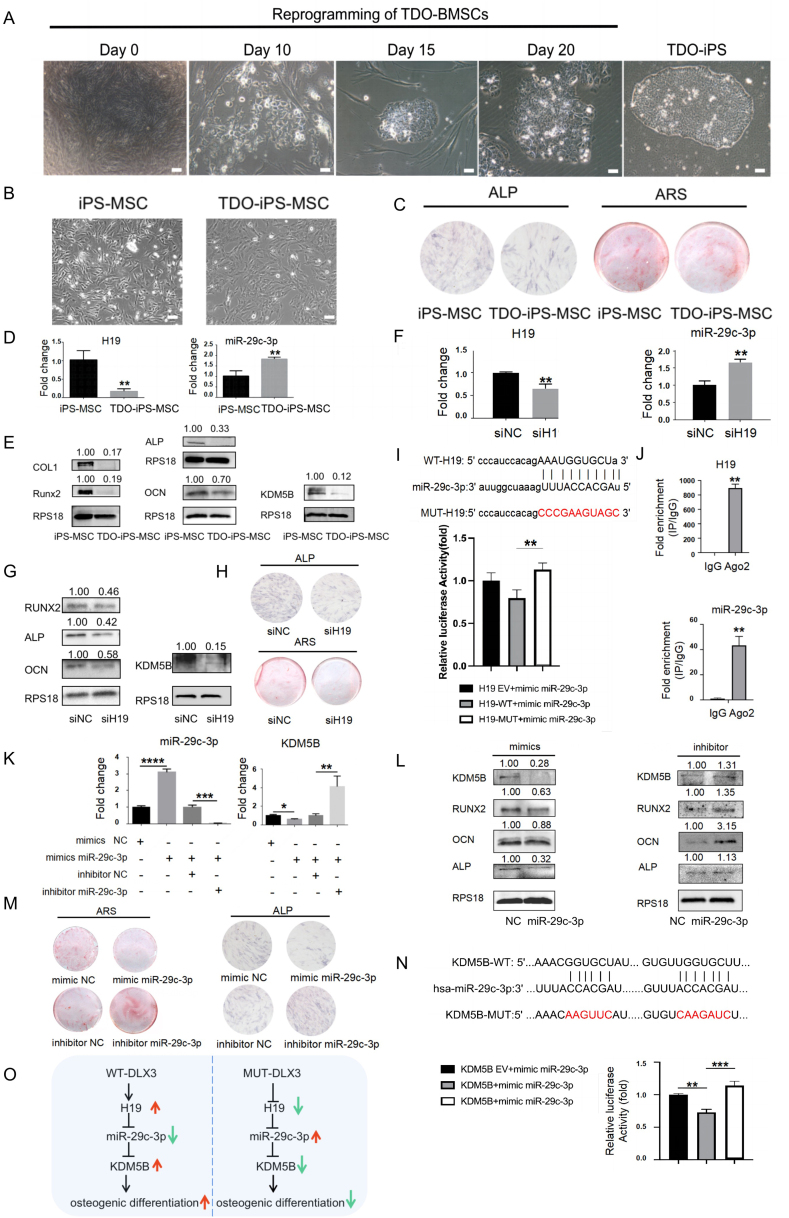
Generation of TDO-iPS and a novel pathway of DLX3(Q178R) mutation delays osteo-differentiation. Generation of iPS cells derived from the BMSCs of a TDO patient. **(A)** Morphological change during the reprogramming of TDO-BMSCs using Sendai viral constructs expressing four transcription factors (OCT4, SOX2, KLF4, and C-MYC). Bar scales: 200 μm for Day 0, 50 μm for Day 10, Day 15, and Day 20, and 100 μm for TDO-iPS. **(B)** iPSCs and TDO-iPSCs were induced to iPS-MSCs and TDO-iPS-MSCs. Bar scales: 100 μm. **(C)** ALP staining and ARS of normal iPS-MSCs and TDO-iPS-MSCs after induced in osteogenic differentiation medium. **(D)** q-PCR analysis of H19 and miR-29c-3p in iPS-MSCs and TDO-iPS-MSCs after 3 days of osteo-differentiation. **(E)** Western blot analysis of osteo-differentiation markers RUNX2, COLI, ALP, OCN, and potential regulatory protein KDM5B in iPS-MSCs and TDO-iPS-MSCs after 3 days of osteo-differentiation. Data are expressed as mean ± SD. ^∗^*P* < 0.05, ^∗∗^*P* < 0.01, ^∗∗∗^*P* < 0.001. **(F)** q-PCR analysis of H19 and miR-29c-3p in TDO-iPS-MSCs transfected with siH19 and osteo-induction culture for 3 days. **(G)** Western blot analysis of osteo-differentiation markers and KDM5B in TDO-iPS-MSCs, cells were transfected with siH19 at 3 days after osteo-differentiation. **(H)** ALP staining and ARS of TDO-iPS-MSCs transfected with siH19 at 7 or 14 days after osteo-differentiation. **(I)** Luciferase assay plasmids were constructed (left) and the results confirmed H19 binds to miR-29c-3p. **(J)** RIP-qPCR analysis of H19 and miR-29c-3p in TDO-iPS-MSCs. Data are expressed as mean ± SD. ^∗^*P* < 0.05, ^∗∗^*P* < 0.01, ^∗∗∗^*P* < 0.001. **(K)** TDO-iPS-MSCs were transiently transfected with mimics (50 nM) or inhibitor miR-29c-3p (100 nM) and their negative controls, respectively, and examined by qPCR, and Western blot analysis **(L)** at 3 days after osteo-differentiation. **(M)** ALP staining and ARS of TDO-iPS-MSCs transfected with mimics or inhibitor miR-29c-3p at 7 or 14 days after osteo-differentiation. **(N)** Luciferase assay plasmids were constructed (left) and the results confirmed KDM5B is the target gene of miR-29c-3p. **(O)** The regulatory relationship of DLX3 and mutant DLX3 regulating osteo-differentiation. Data are expressed as mean ± SD. ^∗^*P* < 0.05, ^∗∗^*P* < 0.01, ^∗∗∗^*P* < 0.001.

We performed immunofluorescence to examine the protein expression of embryonic stem cell markers in the TDO-iPS cells. We showed that these cells expressed TRA-1-61, OCT4, SSEA-4, NANOG, TRA-1-81, and SOX2 (Fig. S2). The results showed that TDO-iPSCs expressed all the above stemness biomarkers, which indicated that TDO-iPSCs may have a similar biological function to stem cells.

To examine the differentiation potential of the TDO-iPS cells *in vivo*, we transplanted the cells into severe combined immunodeficiency mice and monitored teratoma formation three months after the transplantation (Fig. S3). Hematoxylin and eosin staining of the teratoma section showed that the TDO-iPS cells could differentiate into the three germ layers *in vivo*. Immunostaining of endoderm biomarker alpha-fetoprotein, mesoderm biomarker α-smooth muscle actin, and ectoderm biomarker β-III tubulin further confirmed the three types of tissues in teratoma (Fig. S3).

To explore the underlying mechanism of DLX3 regulating osteogenic differentiation, we induced normal iPSCs and TDO-iPSCs to iPS-MSCs and TDO-iPS-MSCs (Fig. 1B). The results of flow cytometry showed that the iPS-MSCs and TDO-iPS-MSCs were successfully generated by detection of mesenchymal biomarkers CD73 (cluster of differentiation 73) and CD146, endothelial cell biomarker CD34, and hematopoietic biomarker CD45 (Fig. S4). The osteogenic capability of TDO-iPS-MSCs was weaker than that of iPS-MSCs according to the results of ALP staining and alizarin red staining (ARS). After incubating in an osteo-differentiation medium for 3 days, we detected the expression of several osteogenic differentiation markers, H19, miR-29c-3p. The results of qPCR (Fig. 1D) and Western blot (Fig. 1E) showed that H19, osteogenic differentiation markers, and KDM5B were down-regulated in TDO-iPS-MSCs while miR-29c-3p was up-regulated. Therefore, we deduced that DLX3 (Q178R) mutation could suppress osteo-differentiation and H19 while promoting miR-29c-3p in iPS-MSCs.

According to the prediction by Starbase (https://starbase.sysu.edu.cn/), H19 could bind to miR-29c-3p (Fig. S5). To further explore the role of H19 in osteo-differentiation, TDO-iPS-MSCs were transiently transfected with H19 siRNA (siH19). The results of qPCR (Fig. 1F), Western blot (Fig. 1G), ALP staining, and ARS staining (Fig. 1H) showed that knockdown of H19 inhibited osteo-differentiation and KDM5B expression. Furthermore, the expression of miR-29c-3p was increased in the siH19 group. We performed RIP-qPCR (Fig. 1J) and luciferase assays (Fig. 1I) to confirm the relationship between H19 and miR-29c-3p. The results showed that the expression of H19 and miR-29c-3p were higher in the anti-Ago2 than IgG control group. Rescue assay was performed by luciferase reporter assay which showed that the Luc/Reni expression level of the WT-H19+miR-29c-3p mimics group was lower than the MUT-H19+miR-29c-3p mimics group. In a word, H19, which was down-regulated by mutant DLX3 and inhibited miR-29c-3p, could promote osteo-differentiation.

Then, we explored the effect of miR-29c-3p in TDO-iPS-MSCs during osteo-differentiation by qPCR (Fig. 1K) and Western blot (Fig. 1L). The results indicated that miR-29c-3p could inhibit osteo-differentiation and KDM5B. As shown in Fig. 1M, the results of ALP and ARS staining also supported the negative effect of miR-29c-3p in osteo-differentiation. According to the predicted results (Fig. 1N) of Targetscan (http://www.targetscan.org/) and Starbase (https://starbase.sysu.edu.cn/), miR-29c-3p could bind to KDM5B 3′UTR. Then, we performed luciferase assays to confirm that miR-29c-3p suppressed KDM5B expression by binding to its 3′UTR. Finally, we did rescue assays by co-transfection of miR-29-3p inhibitor and siKDM5B to verify the relationship between miR-29c-3p and KDM5B. The results of the rescue assay (Fig. S6) showed that miR-29c-3p inhibitor promoted osteo-differentiation and siKDM5B could reverse the increase caused by miR-29c-3p inhibitor. Thus, miR-29c-3p suppressed osteo-differentiation while KDM5B promoted osteo-differentiation. Based on the above results, DLX3(Q178R) inhibited osteo-differentiation via H19/miR-29c-3p/KDM5B axis (Fig. 1O).

TDO-derived iPSCs preserved the genetic characteristics of the host and the iPSCs could be a potential resource for bone tissue regeneration. Here we generated TDO-derived iPS cells, which could be a useful tool for the purpose. As far as we know, it is the first time to generate iPSCs derived from TDO patients in the world. Compared with BMSCs, the stemness and differentiation potential are better in MSCs derived from iPSCs. iPSCs are also a great cell model to investigate the regulatory mechanism of mutant DLX3 regulating tooth and hair follicle formation *in vitro*. In tissue engineering, iPSCs are a great tool that could differentiate into other cells or tissue for repair. To date, how to control iPSCs' differentiation into the cells we expected is still a challenge.

In conclusion, we successfully established TDO-iPSCs carrying the genetic mutation of the host cells, which may be a useful tool for studying the molecular mechanism of TDO syndrome and accelerating the translation research of TDO-iPSCs based on TDO patients' favorable bone characterization. DLX3(Q178R) suppresses osteogenesis via H19/miR-29c-3p/KDM5B axis *in vitro* (Fig. S7)*.* The research of DLX3(Q178R) mutation regulating osteogenesis provides a novel possibility.

## Ethics declaration

This study was approved by the Committee of the Peking University School of Stomatology (No. LA2015021).

## Author contributions

Conceptualization: ZM, CD, and YW. Data curation and analysis: LD, NZ, and DW. Validation and methodology: MW, YZ, and LS. Writing-original draft: LD, NZ, and DW. Writing-reviewed draft: ZM, CD, and YW. Approval of the final draft: all authors.

## Conflict of interests

The authors have no competing interests to declare.

## Funding

This study was supported by the National Nature Science Foundation of China (No. 81970920 and 81900983), the Natural Science Foundation of Beijing Municipality, China (No. 7232218), and the Shanghai Science and Technology Committee Youth Sailing Program (China) (No. 19YF1442500).

## References

[1] Price J.A., Wright J.T., Kula K., Bowden D.W., Hart T.C. (1998). A common *DLX3* gene mutation is responsible for tricho-dento-osseous syndrome in *Virginia* and North *Carolina* families. J Med Genet.

[2] Shapiro S.D., Quattromani F.L., Jorgenson R.J., Young R.S. (1983). Tricho-dento-osseous syndrome: heterogeneity or clinical variability. Am J Med Genet.

[3] Zhao N., Han D., Liu H. (2016). Senescence: novel insight into DLX3 mutations leading to enhanced bone formation in Tricho-Dento-Osseous syndrome. Sci Rep.

[4] Liu Y., Zhou Y. (2022). Circ_0087960 stabilizes KDM5B by reducing SKP_2_ mediated ubiquitination degradation and promotes osteogenic differentiation in periodontal ligament stem cells. Regen Ther.

