# Supplementary material for: DLX3 (Q178R) mutation delays osteogenic differentiation via H19/miR-29c-3p/KDM5B axis in TDO-iPSCs-derived MSCs
Source: Genes Dis. 2023 Jul 3;11(4):101012. doi: 10.1016/j.gendis.2023.05.011 (PMC10933465; doi:10.1016/j.gendis.2023.05.011)
Supplement: Multimedia component 1 [file mmc1.doc]

**Supplementary Materials**

**Introduction**

Tricho-Dento-Osseous (TDO) syndrome is a rare autosomal dominant disease mainly characterized by kinky hair, enamel and dentin hypoplasia and accumulating increased the bone volume and density of skull and mandible^1; 2^. A single gene, distal-less homeobox 3 (DLX3), mutations are identified as the TDO-causing gene, which is mapped to 17q21.33, and plays a crucial function in the development and differentiation of hair, tooth and bone^3^. Up to this point, 6 mutations of DLX3 were found in TDO patients in the whole world and all mutations locate at the functional domains^3-7^. The mutations are listed in Table I. Affected people mainly suffer from hair and tooth problems. They have to wear wig and denture to solve these clinical problems. TDO-associated accumulating increased the bone volume and density of skull is not a complaint of the affected patients.

Table I Reported DLX3 mutant types in TDO syndrome

| **DLX3 mutation type** | **Affected exon and domain** | **Typical clinical features** |
| --- | --- | --- |
| c.398G>C, R133P | Exon 2; Homeodomain | A Finnish family with typical TDO syndrome features in bone, tooth and hair^7^ |
| c.524T>C, I175T | Exon 2; Homeodomain | Sporadic case with kinky hair, enamel defects and atopic dermatitis in Austria |
| c.533A>G, Q178R | Exon 3;  Homeodomain | Sporadic case with TDO syndrome features in China^5^ |
| c.545C>T, S182F | Exon 3;  Homeodomain | A Finnish family with lanugo-type hair and severe dental abnormalities^7^ |
| c.561-562 delCT, A>G, Y188QfsX13 | Exon 3;  Homeodomain | an Australian family with hypoplastic-hypomaturation-type amelogenesis imperfecta with taurodontism (AIHHT) ^8^  a Korean family overlapping an attenuated phenotype of TDO and AIHHT^4^ |
| c.517-574 delGGGG, G191RfsX66 | Exon 3;  Homeodomain | In Virginia and North Carolina families with TDO syndrome ^1; 2^ |

Previous *in vitro* and *in vivo* studies show that DLX3 is required for hair, tooth, bone and placental development^8-10^. Inactivation of *Dlx3* leads to death owing to placental failure in mice. Transgenic mice with DLX3 (c.533A>G, Q178R) mutation present the delayed bone senescence process of BMSCs, and *in vitro* high growth rate of BMSCs^11^.

To date, the underlying mechanisms regarding how DLX3 regulates the development of hair, tooth and bone remain largely unknown. The animal models are not exactly identical with TDO syndrome in human. Using human cells from TDO patient is necessary to investigate the mechanism of TDO. However, getting the cells from TDO patients is a rare chance. Fortunately, with informed consent and proven by the Ethics Committee of Peking University School of Stomatology, we got a tiny alveolar bone tissue from the TDO patient who possesses DLX3 (c.533A>G, Q178R) mutation and needed to do alveoloplasty and implantation, and then isolated BMSCs. Considering that the limited number of primary TDO BMSCs is not enough to investigate the underlying mechanisms, we were planning to generate the induced pluripotent stem (iPS) by using the primary TDO BMSCs to solve the problem. Human somatic cells can be reprogrammed into iPS and acquire a nearly unlimited capacity of self-renewal and pluripotency by introducing a set of transcription factors including OCT-4, SOX-2, KLF4 and c-MYC^12^.

According to our group previous studies, mutant DLX3(Q178R) could regulate lncRNAs and miRNAs in many physiological and pathological processes. Long non-coding RNAs (lncRNAs) are a kind of non-coding transcripts with the lengths of greater than 200 nucleotides^13^. H19 is a famous long-noncoding RNA (lncRNA) which has been verified to participate in many diseases, especially in cancers^14; 15^, and physiology processes, including proliferation, apoptosis^16^, differentiation^17^ and so on. The ways of H19 performing its function are various, it could be regulated by [histone modification](javascript:;)^18^ like H3K4me3 and H3K27me3^19^, and also regulate miRNAs though ceRNA networks^20^. Most of the previous studies favored that H19 promotes osteogenic differentiation through lncRNA-miRNA pathway^21^. Our previous studies also demonstrated that H19 could be regulated by mutant DLX3 and influence BMSCs osteogenic differentiation^9^ and Mut-DLX3 increases methylation of H19 promoter in hDPC was due to the changes in DNMT3B activity^8^. [With the deepening of research](javascript:;)es of non-coding RNA, it has been demonstrated that lncRNA and miRNA play [crucial part in](javascript:;) physiological or pathological activities^22^. According to existing studies, microRNA could be sponged by lncRNA to regulate osteogenic differentiation^23^. We noticed that H19 could regulate miR-29 family, which includes 3 main members, miR-29a^24^, miR-29b^25^ and miR-29c^26; 27^. miR-29c-3p has been verified that could negatively regulate osteoblasts differentiation of rat BMSCs in hyperlipidemia environment by targeting Dvl2^28^. Furthermore, inhibition of miR-29-3p isoforms via tough decoy could promote intermittent parathyroid hormone-induced bone anabolism^29^. KDM5B has also been demonstrated to bind to promoter region of alkaline phosphatase (ALP), RUNX family transcription factor 2 (RUNX2) and osteocalcin (OCN)^30^, which are crucial in osteogenesis. However, the mechanism by which H19 regulates osteogenic differentiation through miR-29c-3p/KDM5B still remains largely unknown.

Our previous reports have been shown that the missense mutation of DLX (Q178R) in a TDO patient is found in our group. The DLX3 (Q178R) mutation affects a conserved residue in the homeodomain DLX3.In this study, we generated TDO-iPS cells derived from a TDO patient-derived mesenchymal stem cells with DLX3 (Q178R, c.533A>G) mutation using Sendai viruses expressing the four transcription factors (OCT4, SOX2, KLF4 and C-MYC). The TDO-iPS cells could provide enough human cells with DLX3 mutation to investigate the underlying mechanism involved in TDO syndrome.

**Materials and Methods**

**1. Human subjects**

Written informed consent was obtained from a TDO patient who visited the Peking University School of Stomatology, and this study was approved by the Ethics Committee of Peking University School of Stomatology (LA2015021).

**2. Cell culture**

A tiny TDO alveolar bone tissue was obtained from a patient with DLX3 (c.533A>G, Q178R) mutation. The fresh tissue was digested with collagenase I for 1h, and the dissociated cells (BMSCs) were then plated on 10-cm tissue culture dishes in Dulbecco’s modified Eagle’s medium (DMEM, Invitrogen, Carlsbad, CA, USA) containing 10 % fetal bovine serum (FBS), 2 mM glutamine (Sigma-Aldrich, Darmstadt, Germany), and antibiotics (100 U/mL penicillin and 100 μg/ml streptomycin, Sigma) (complete medium) at 37 °C in a humidified atmosphere containing 5 % CO_2_.

**3. iPS cell generation**

Sendai virus containing OCT4, SOX2, KLF4, and c-MYC were used to generate iPS cells. BMSCs from the TDO patient (TDO-BMSCs) were incubated in recombinant Sendai virus-containing medium for 24 h, and then changed with complete medium every day. Seven days later, the cells were harvested by trypsinization, and 5 × 10^4^ cells were placed on Matrigel coated dishes in Reproeasy medium (Cellapybio Biotechnology Co. Ltd, Beijing, China) supplemented with 4 ng/mL bFGF. The medium was changed daily until iPS clones with morphological changes were monitored.

**4. MSC cell generation and osteogenic differentiation**

Normal human iPS (purchased from Beijing Cellapy Biotech Co.) and TDO-iPS were redifferentiated toward iPS-derived MSCs (hereafter named iPS-MSCs) and TDO-iPS-derived MSCs (hereafter called TDO-iPS-MSCs) according to the manufacturer’s instructions of STEMdiff™ Mesenchymal Progenitor Kit (STEMCELL Technologies, Vancouver, BC, Canada). Briefly, the mTeSR™1 medium was replaced by 3 mL STEMdiff™-ACF mesenchymal induction medium for 3 days, and exchanged the medium every day. Then the cells were cultured with MesenCult-ACF Plus medium up to 21 days until iPS-MSCs and TDO-iPS-MSCs were successfully induced. The DLX3 mutation in TDO-BMSCs and TDO-iPS cells were detected by the PCR product of DLX3 genomic DNA. PCR reaction was performed by PCR GreenMaster Mix with 50 ng of DNA and DLX3 primer pair. The primer sequences were as described in Kim *et al*^31^. The sequencing primer for DLX3 PCR product is 5’-GCATTCTGAGAGGCTAACTAGCTAC-3’.

For detect the multi-differentiation potential iPS-MSCs and TDO-iPS-MSCs were cultured into osteogenic differentiation medium respectively. The osteogenic differentiation medium (ODM) is MSCM (Hyclone，Logan, Utah, USA) supplementing 10 mM sodium β-glycerophosphate, 50 μM ascorbic acid and 0.1 μM dexamethasone (Sigma-Aldrich), 100 U/mL penicillin, and 100 µg/mL streptomycin The iPS-derived cells were incubated in osteogenic medium and myogenic medium respectively for 21 days. The medium was changed every 3 days Alkaline phosphatase (ALP) staining （Beyotime, Shanghai, China）was performed on day 7 and Alizarin Red staining (Cyagen Biosciences, Guangzhou, China)was performed on day 21 to detect the osteogenic differentiation potential of iPS-derived cells. All cells were cultured at 37^◦^C in a 5% CO_2_, 100% humidity incubator.

**5. Transient transfection**

1×10^5^ cells per well were inoculated into 6-well plates with 2 mL medium for overnight. Transfection assay was carried out using Lipo8000 reagent (Beyotime, Shanghai, China) with plasmids or miR-29c-3p mimics or their hairpin inhibitors (RiboBio, Guangzhou, China) according to the manufacturer’s instructions. At 24h post-transfection, the culture medium was replaced by fresh osteogenic differentiation medium. Cells were incubated in osteogenic differentiation medium for 3 days and harvested to detect osteogenic genes expression at mRNA and protein levels by real-time PCR and western blot, respectively.

**6. Karyotype analysis**

TDO-iPS cells were treated with 50 ng/mL demecolcine for 6 h when TDO-iPS cells reached 80% confluence. The cells were detached by EDTA, and single cell suspension was harvested by centrifuge at 1000 rpm for 10 min, washed once with PBS, treated with low permeability KCL solution for 40 min at 37°C. Then the cells were fixed by the mixture of methanol and acetic acid glacial at the ratio of 3:1 at 4°C overnight. At the following day, the cells were subjected to G-band staining and karyotype analysis.

**7. Real-time PCR**

Total RNAs derived from TDO-BMSCs and TDO-iPS cells were extracted by using TRIzol® reagent (Invitrogen Life Technologies, Grand Island, NY, USA) and reverse-transcribed into cDNA using the Superscript first-strand synthesis system (Promega, Madison, WI, USA) according to manufacturer’s protocol. miRNA was reverse-transcribed using the miDETECT A Track™ miRNA qPCR kit (RiboBio, Guangzhou, China) following the manufacturers’ instructions. Real-time PCR reactions were conducted to analyze the expression of mRNA and miRNA in a real-time PCR system (Abclonal, Wuhan, China) using GoTaq® Green Master Mix (Promega, Madison, WI, USA) in a 10 μl reaction mixture. The mRNA expressions were normalized to RPS18 and miRNA expressions were normalized to U6, both are using the 2^-∆∆^ Ct method. miRNA primers were purchased from Guangzhou RiboBio Co., Ltd (RiboBio). The real-time PCR primers were listed in Table II.

Table II Primer sequences using in this study

| Gene | Forward（5’-3’） | Reverse（5’-3’） |
| --- | --- | --- |
| RPS18 | GCGGAAAATAGCCTTTGCCAT | TGATCACACGTTCCACCTCAT |
| H19 | GCACCTTGGACATCTGGAGT | TTCTTTCCAGCCCTAGCTCA |
| KDM5B | CCATAGCCGAGCAGACTGG | GGATACGTGGCGTAAAATGAAGT |
| U6 | CTCGCTTCGGCAGCACA | AACGCTTCACGAATTTGCGT |

**8. Western Blot**

We harvested protein by using RIPA buffer (Beyotime) with protease inhibitors (Roche Diagnostics). Each sample was separated by SDS-PAGE gel (Biotides, Beijing, China) and transferred to a polyvinylidene difluoride membrane. After blocked for 1 h in 5% nonfat milk at room temperature, the membranes were incubated in antibodies against DLX3 (1:1000 dilution, Abcam, Cambridge, UK), KDM5B (1:1000 dilution, Abclonal, Wuhan, China), OCN (1:1000 dilution, Abclonal), RUNX2 (1:1000 dilution, Proteintech, Rosemount, IL, USA) and RPS18 (1:1000 dilution, Abclonal, Wuhan, China) overnight at 4 °C. Then the membranes were incubated with HRP-conjugated secondary antibodies (1:10000, Huaxingbio, Beijing, China) for 1 h at room temperature and incubated with BeyoECL Plus (Beyotime). The quantification of results was made by ImageJ software.

**9. Immunofluorescence**

The TDO-iPS cells were washed twice with phosphate-buffered saline (PBS), fixed with 4 % (w/v) fresh-made paraformaldehyde for 20 min, permeabilized for 60 min with PBS containing 0.1 % (v/v) Triton X-100, and then blocked for 3 h with PBS containing 5 % BSA. The fixed samples were incubated with anti-human TRA-1-60, anti-human OCT4, anti-human SSEA-1, anti-human TRA-1-81 and anti-human SOX2 antibodies (all from CST Systems) as indicated, and then washed three times with PBS containing 0.1 % (v/v) Triton X-100 and probed with fluorescein-labeled the appropriate secondary antibodies. Nucleus were counter-stained with 4',6-diamidino-2-phenylindole (DAPI).

**10. *In vivo* pluripotency assessment**

TDO-iPS cells (1×10^7^) were subcutaneously injected into the back of 5-week-old SCID mice. Three months after injection, the mice were sacrificed for examination of teratoma formation. The identified tumors were dissected, fixed and subjected to pathological analyses. Immunohistochemistry of fetoprotein, α-SMA and βIII-tubulin for evaluation of endodermal, mesodermal and ectodermal tissues, respectively.

**11. Immunohistochemistry**

The TDO-iPS-derived teratoma tissues were sectioned (5 μm) for immunohistochemistry as previously described^32^. Primary antibody against α-fetoprotein, α-smooth muscle actin (α-SMA), and βIII-tubulin were used as three-layer biomarkers to investigate the endodermal, mesodermal and ectodermal differentiation, respectively.

**12. Alkaline phosphatase (ALP) staining**

Cells after infected were cultured in 24-well plates in osteo-differentiation medium (ODM) for 7 days and performed by ALP staining using the NBT/BCIP staining kit according to the manufacturer's protocol (Beyotime).

**13. Alizarin red staining (ARS)**

Cells after infected were induced by ODM in 24-well plates for 3 weeks. ARS were performed according to the manufacturer's protocol.

**14. Luciferase reporter Assay**

We did luciferase reporter assays in HEK293T cells by the indicated co-transfection of pGL3B empty vector, pGL3B-KDM5B-wt or pGL3B-KDM5B-mut and 50 nM miR-29c-3p mimics or mimics control oligonucleotides (NC) (RiBoBio) with the pTK-RL plasmid carrying the Renilla luciferase expression cassette, respectively. Luciferase assays were performed according to the manual of the Dual Luciferase Reporter Gene Assay Kit (Beyotime).

**15. Flow cytometry analysis**

Cells were digested by trypsin (Gibco, Carlsbad, CA, USA) to single cell and stained with antibody for 30 min at 4 °C. Then analyzed by Novocyte (Agilent, Santa Clara, California, USA). The antibodies were as follows: FITC anti-human CD73 (Biolegend, San Diego, CA, USA), FITC anti-human CD146 (Biolegend), FITC anti-human CD34 (Biolegend) and FITC anti-human CD45 (Biolegend).

**16. RNA immunoprecipitation (RIP) assay**

To determine the relationship between H19 and miR-29c-3p, a RIP RNA-Binding Protein Immunoprecipitation Kit (Gzscbio, China) was used and the extracted RNAs were used to evaluated by RT-PCR. Antibodies used for the RIP assay included anti-AGO2 (Abclonal) and control IgG.

**17. Statistical analysis**

All data was presented as mean±SD of three independent experiments. Statistical significance was determined by one-way analysis of variance, followed by Tukey’s post-hoc test when there are more than 2 groups, or t test when there are only 2 groups; and *P* < 0.05 was set statistically significant.

**Discussion**

Patient-specific iPSCs with self-renewal and pluripotent abilities can yield a large variety of patient cells as the important cell source to investigate specific tissues/organs development, establish disease models, and develop a new treatment approach.

TDO syndrome is a single gene DLX3 mutation caused skull, teeth and hair mal-developmental disease^1; 3^. It is a good model for investigating the role of DLX3 in bone, tooth and hair development and the related molecular signaling involved. A better understanding of the mechanism is required for the development of a new treatment approach for TDO patients. Here we generated TDO-derived iPS cells, which could be a useful tool for the purpose. As far as we know, it is the first time to generate iPS derived from TDO patients in the world. There are many methods to generate iPS using viral vectors expressing OCT4, SOX2, KLF4, and c-MYC^33^. In this study, we chose the Sendai virus as vector because the virus not only efficiently induces iPS cells, but also does not integrate into the host genome, finally reducing the potential tumorigenicity of the iPS cells, and increasing the potential applications as biomedicine^34^. Immunofluorescence assay showed that TDO-iPS cells could express stem cell markers^35^ including TRA-1-61, OCT4, SSEA-4, NANOG, TRA-1-81 and SOX2, which indicated that TDO-iPS was successfully established. DNA sequencing showed TDO-derived iPS cells and TDO-derived BMSCs had the same DLX3 (c.533A>G, Q178R) mutation and normal karyotype, which suggested that TDO-derived iPS preserved the genetic characteristics of the host and the iPS could be used to investigate the molecular mechanism of TDO syndrome.

The distinguishing features of iPS are pluripotent to differentiate into several types of cells *in vitro* and form teratomas with different tissues *in vivo*. In this study, we verified TDO-derived iPS could differentiate into osteogenic lineage cells with potential osteogenesis capacity under osteo-induction stimulation, and form teratoma after TDO-iPS were subcutaneously inoculated into the back of 5-week-old SCID mice. Pathologic examination clearly showed that TDO-iPS derived teratoma had the three germ layers (α-AFP for endoderm^36^, α-SMA for mesoderm^37; 38^ and β-III tubulin ectoderm^39^). These results indicated that TDO-iPS had multi-potent ability and preserve the characteristics of host cells. Considering the facts that: ①TDO patients with DLX3 mutation present cumulatively increased bone density in skull and alveolar bone; ②Transgenic mice with DLX3 (c.533A>G, Q178R) mutation present higher bone density through delaying the bone senescence process of BMSCs, and increasing BMSCs cell number, the TDO-derived iPS cells have not only the extremely high research value in bone, tooth and hair biology, but also have potential value in clinical practice for anti-bone ageing and increase in bone density in future.

Since DLX3 is essential in bone formation, iPSCs is a great cell model to explore the mechanism of DLX3 regulating osteogenic differentiation. We induced TDO-iPS to TDO-iPS-MSCs and designed experiments *in vitro*. Our group demonstrated that mutant DLX3 could suppress proliferation *via* down-regulation of H19 in BMSCs^9^. Since the changes are opposite *in vivo* and *in vitro*, there is still a gap in researching the function of mutant DLX3 during osteogenic differentiation. Instead of BMSCs, we used MSCs induced from TDO-iPSCs to investigate the underlying mechanism and explored a novel pathway of mutant DLX3 regulating osteogenesis. Firstly, we detected the expression of H19, miR-29c-3p, KDM5B and crucial osteogenesis markers in iPS-MSCs and TDO-iPS-MSCs at both mRNA and protein levels after got the induced MSCs from iPSCs. To confirm the regulated pathway, we knocked down H19, and then detected the [osteogenic capability](javascript:;) by ALP staining and ARS, and the mRNA and protein expression of osteogenic differentiation markers, miR-29c-3p and KDM5B by real-time PCR and western blot. The results of RIP assay and luciferase assay confirmed the relationship between H19 and miR-29c-3p. Obviously, H19 positively regulated osteogenesis and KDM5B and negatively regulated miR-29c-3p. The regulation of miR-29c-3p and KDM5B was confirmed by luciferase assays. miR-29c-3p bound to the 3’UTR of KDM5B to degrade KDM5B and then inhibited osteogenesis. We designed rescue assay to verify the function of miR-29c-3p and KDM5B on osteogenic differentiation. Down-regulated miR-29c-3p could promote osteogenesis and knockdown KDM5B could “rescue” the osteogenic effect of miR-29c-3p knockdown. In this study, we explored a novel pathway of mutant DLX3 regulated osteogenesis *in vitro.* DLX3(Q178R) suppressed H19, H19 sponged to miR-29c-3p to reduce its expression, and miR-29c-3p bound to 3’UTR of KDM5B to inhibit KDM5B, which binds to promoter region of ALP, RUNX2 and OCN and inhibits their function, and finally suppress osteogenesis. Our studies provided great support that mutant DLX3 inhibits osteogenesis *in vitro.*

As we described before, H19 also has intimate connection with histone modification. KDM5B, a histone lysine demethylase, catalyzes the demethylation of histone 3 lysine 4 (H3K4)^40^ and histone 3 lysine 9 (H3K9)^41^. The role of KDM5B as a histone lysine demethylase during osteogenic differentiation is still not enough. The relationship between histone modification and mutant DLX3 regulated osteogenesis requires further research.

According to previous studies, miR-29c-3p could reduce bone loss in rat^42^, repress osteogenic differentiation of rat BMSCs in hyperlipidemia environment^28^ and suppress intermittent parathyroid hormone-induced bone anabolism^29^. Our results also support the down-regulation of miR-29c-3p towards osteogenesis. Furthermore, miR-29 family has been verified to take part in regulation of senescence^43^. Since miR-29c-3p was considered to promote senescence^44^, it may be a crucial factor to investigate the difference of mutant DLX3 regulated osteogenic capability between *in vivo* and *in vitro.* However, the regulation of ceRNA is complex, more research is required.

**
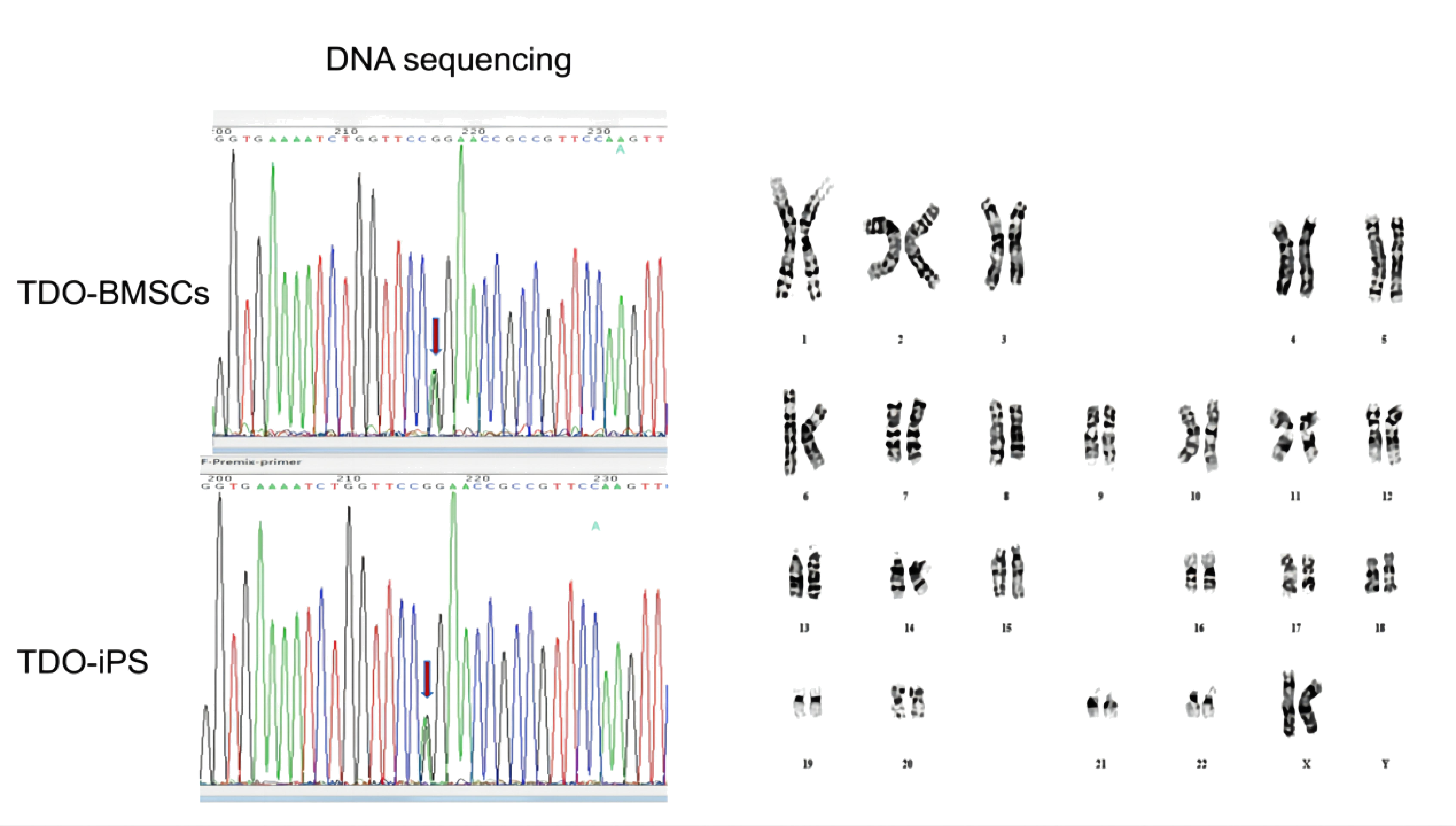
**

Figure S1. Genotype examination and karyotype analysis. DNA sequencing showing TDO-BMSCs and TDO-iPS have the same DLX3 gene mutation. Karyotype analysis showing TDO-iPS cells have normal diploid karyotype with 46, XX.


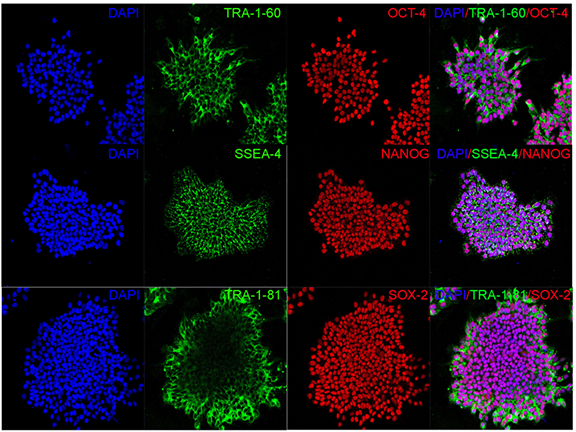

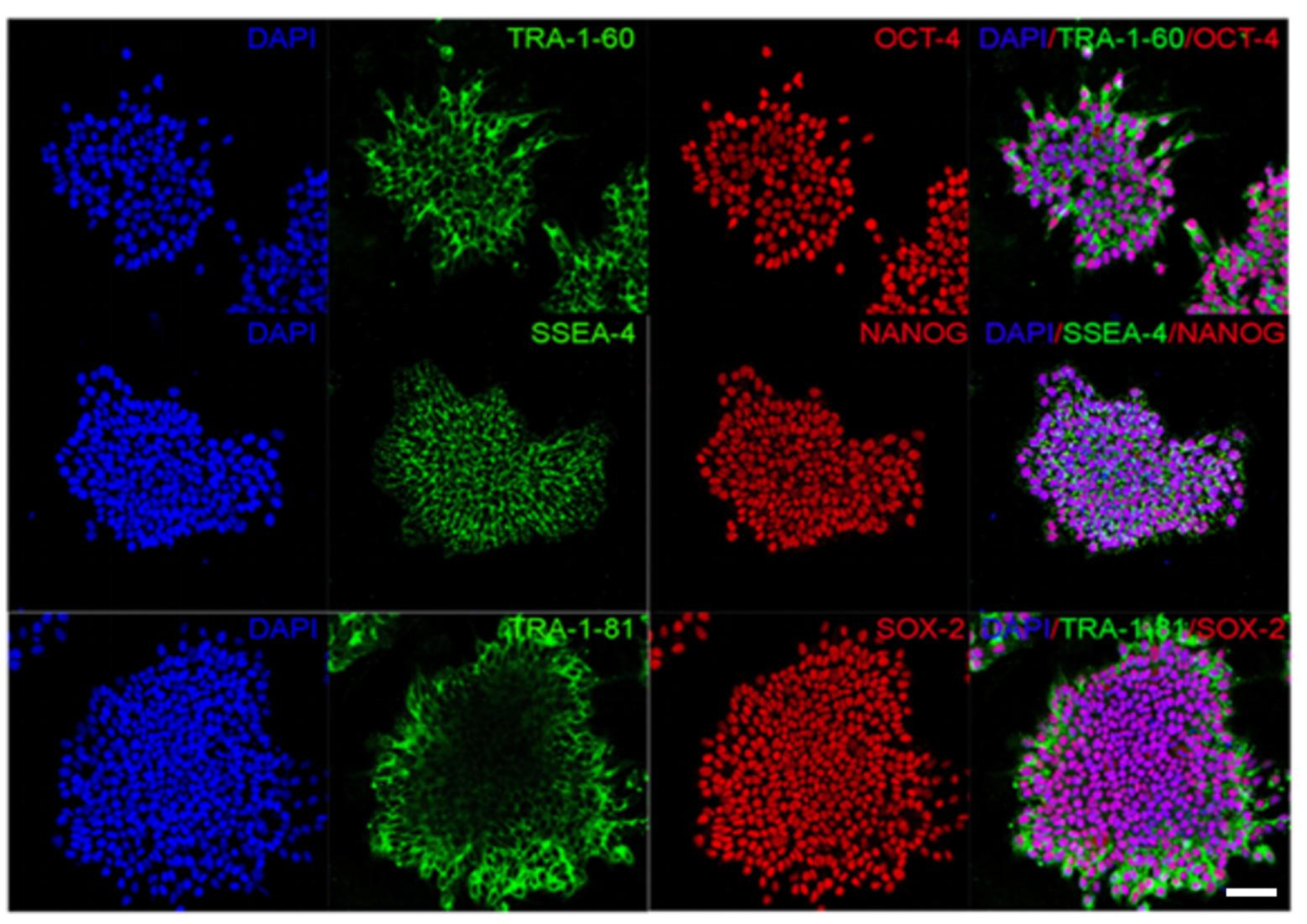


Figure S2. Immunofluorescence staining analysis of the TDO-iPS cells. Nuclei were stained with DAPI with blue fluorescence. Stemness biomarkers TRA-1-60, SSEA-4 and TRA-1-80 were stained by green fluorescence, and stemness biomarkers OCT4, NANOG and SOX2 were stained by red fluorescence. Bar=100 μm.





Figure S3. Teratoma formation after subcutaneously transplantation of the TDO-iPS cells in SCID mice.  HE staining and immunostaining of the TDO-iPS-derived teratomas’ sections. Immunostaining of endoderm biomarker α-AFP, mesoderm biomarker α-SMA and ectoderm biomarker β-III tubulin were confirmed by the pathological examination of the teratoma.


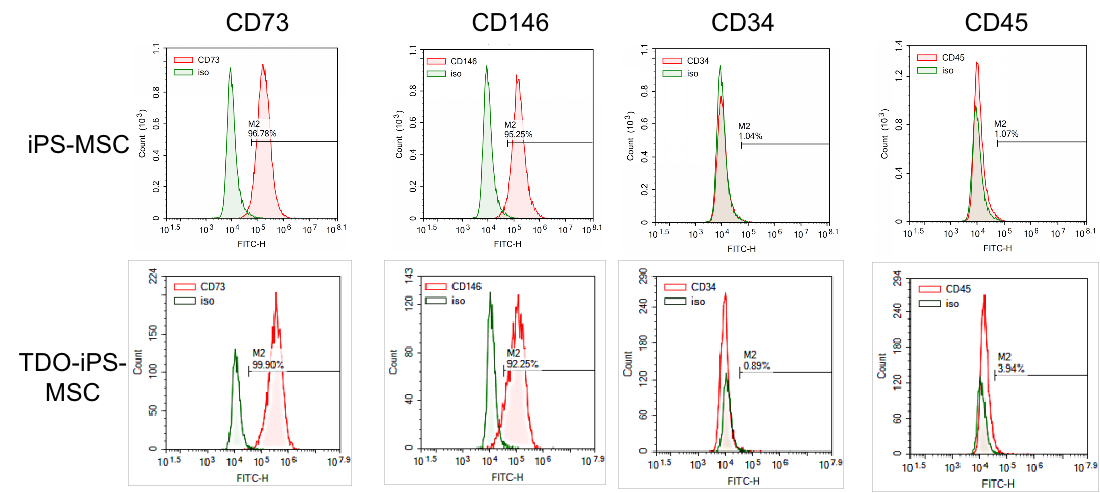


Figure S4. iPS-MSCs and TDO-iPS-MSCs were examined stemness marker by flow cytometry.


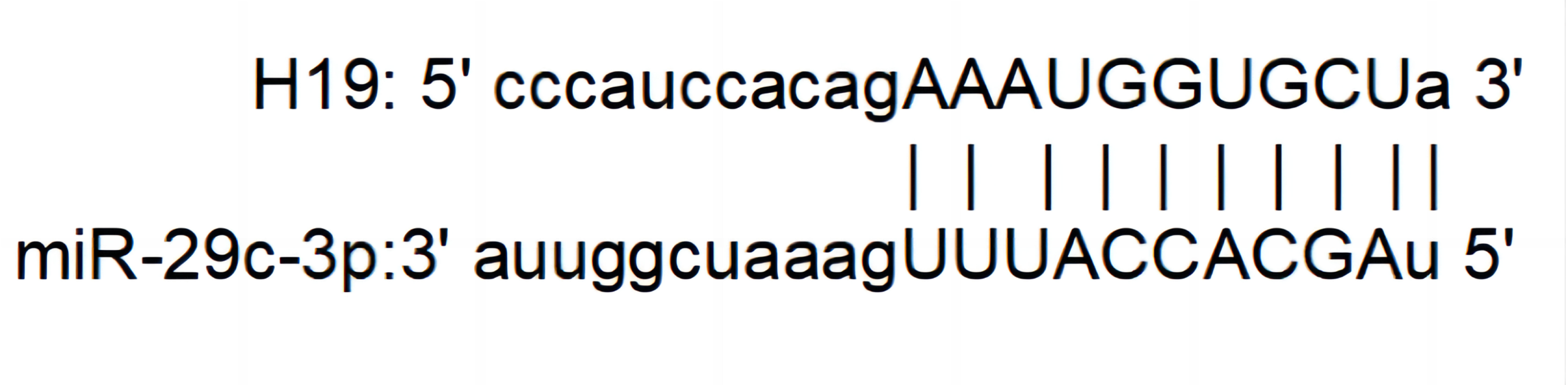


Figure S5. The prediction of H19 binds to miR-29c-3p by Starbase.


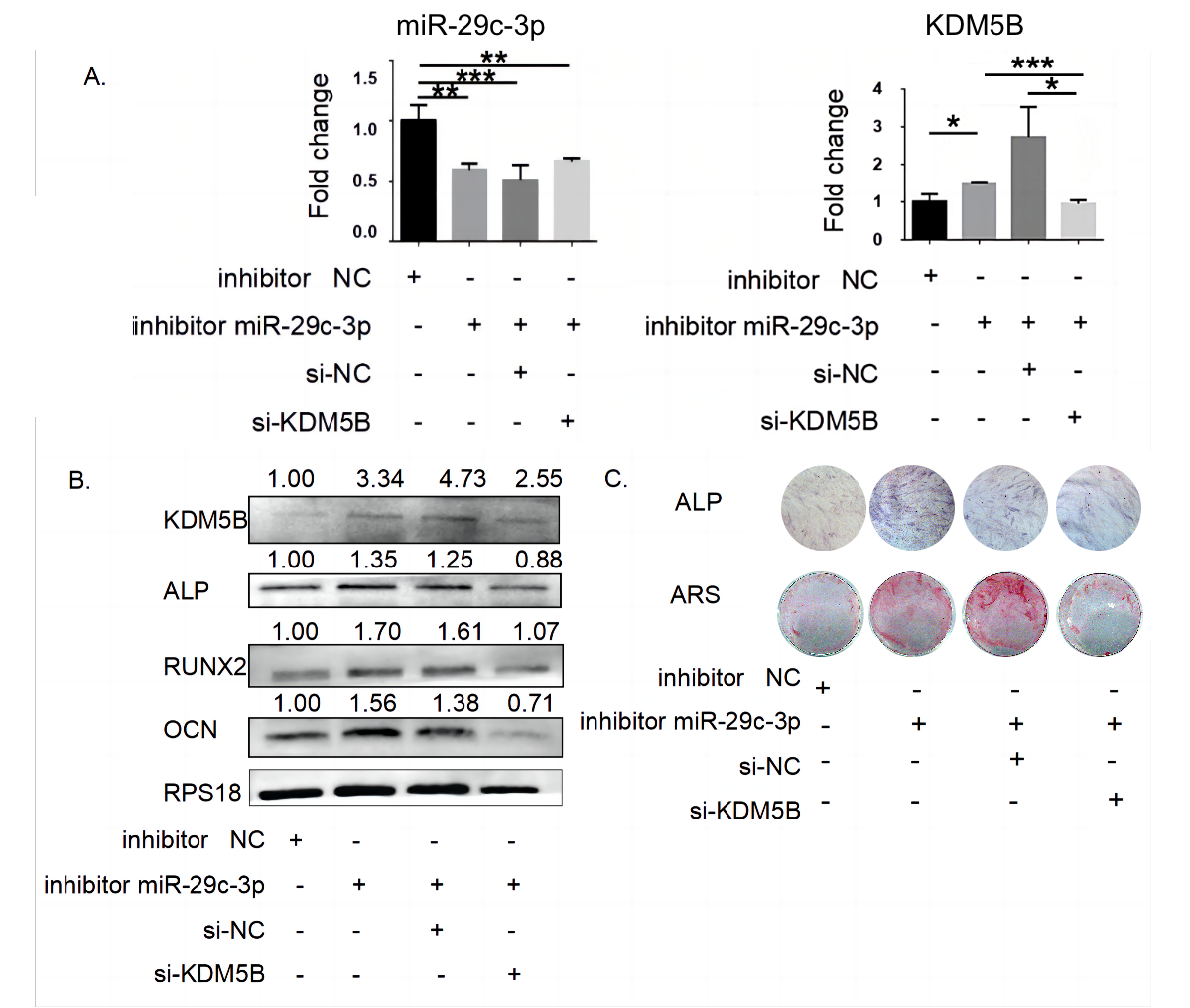


Fig. S6 Rescue assay confirms the relationship between miR-29c-3p and KDM5B by co-transfection of miR-29-3p inhibitor and siKDM5B plasmids and examined by qPCR (A), western blot analysis (B) and ALP staining and ARS (C) after osteogenic differentiation.


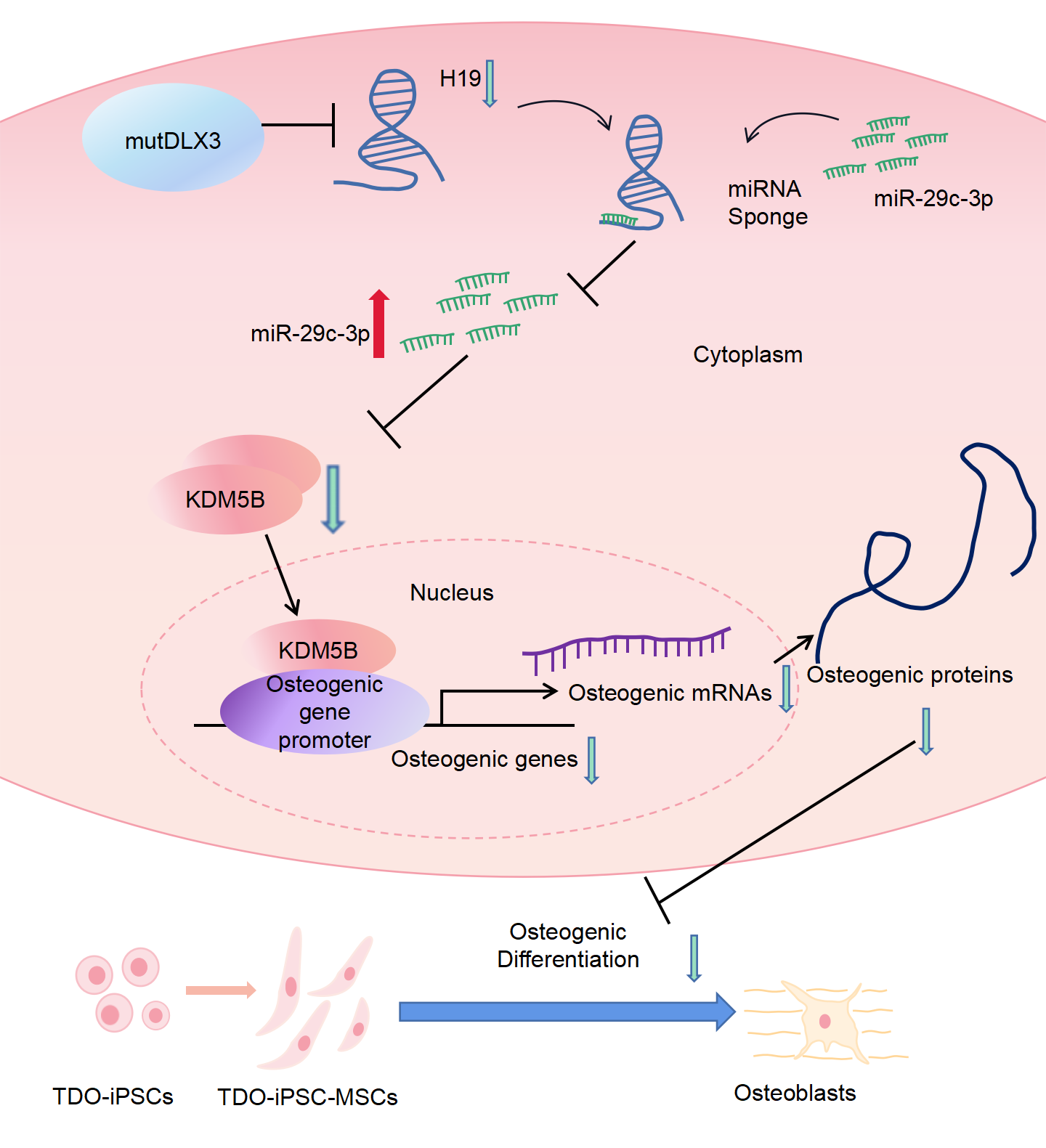


Fig. S7 The mechanism of DLX3(Q178R) mutation delays osteogenic differentiation *via* H19/miR-29c-3p/KDM5B axis in TDO-iPSCs-derived MSCs.

**References**

1. Price JA, Wright JT, Kula K, Bowden DW & Hart TC. A common DLX3 gene mutation is responsible for tricho-dento-osseous syndrome in Virginia and North Carolina families. *J MED GENET* 1998; **35**(10)**:** 825-828.

2. Shapiro SD, Quattromani FL, Jorgenson RJ & Young RS. Tricho-dento-osseous syndrome: heterogeneity or clinical variability. *Am J Med Genet* 1983; **16**(2)**:** 225-236.

3. Price JA, Bowden DW, Wright JT, Pettenati MJ & Hart TC. Identification of a mutation in DLX3 associated with tricho-dento-osseous (TDO) syndrome. *HUM MOL GENET* 1998; **7**(3)**:** 563-569.

4. Lee SK, Lee ZH, Lee SJ, Ahn BD, Kim YJ & Lee SH *et al.*. DLX3 mutation in a new family and its phenotypic variations. *J DENT RES* 2008; **87**(4)**:** 354-357.

5. Li Y, Han D, Zhang H, Liu H, Wong S & Zhao N *et al.*. Morphological analyses and a novel de novo DLX3 mutation associated with tricho-dento-osseous syndrome in a Chinese family. *EUR J ORAL SCI* 2015; **123**(4)**:** 228-234.

6. Mayer DE, Baal C, Litschauer-Poursadrollah M, Hemmer W & Jarisch R. Uncombable hair and atopic dermatitis in a case of trichodento-osseous syndrome. *J DTSCH DERMATOL GES* 2010; **8**(2)**:** 102-104.

7. Dong J, Amor D, Aldred MJ, Gu T, Escamilla M & MacDougall M. DLX3 mutation associated with autosomal dominant amelogenesis imperfecta with taurodontism. *AM J MED GENET A* 2005; **133A**(2)**:** 138-141.

8. Zeng L, Sun S, Dong L, Liu Y, Liu H & Han D *et al.*. DLX3 epigenetically regulates odontoblastic differentiation of hDPCs through H19/miR-675 axis. *ARCH ORAL BIOL* 2019; **102:** 155-163.

9. Zhao N, Zeng L, Liu Y, Han D, Liu H & Xu J *et al.*. DLX3 promotes bone marrow mesenchymal stem cell proliferation through H19/miR-675 axis. *Clin Sci (Lond)* 2017; **131**(22)**:** 2721-2735.

10. Hwang J, Mehrani T, Millar SE & Morasso MI. Dlx3 is a crucial regulator of hair follicle differentiation and cycling. *DEVELOPMENT* 2008; **135**(18)**:** 3149-3159.

11. Zhao N, Han D, Liu H, Li Y, Wong SW & Cao Z *et al.*. Senescence: novel insight into DLX3 mutations leading to enhanced bone formation in Tricho-Dento-Osseous syndrome. *Sci Rep* 2016; **6:** 38680.

12. Eglen RM & Reisine T. Human iPS Cell-Derived Patient Tissues and 3D Cell Culture Part 2: Spheroids, Organoids, and Disease Modeling. *SLAS TECHNOL* 2019; **24**(1)**:** 18-27.

13. Nair L, Chung H & Basu U. Regulation of long non-coding RNAs and genome dynamics by the RNA surveillance machinery. *Nat Rev Mol Cell Biol* 2020; **21**(3)**:** 123-136.

14. Ren J, Ding L, Zhang D, Shi G, Xu Q & Shen S *et al.*. Carcinoma-associated fibroblasts promote the stemness and chemoresistance of colorectal cancer by transferring exosomal lncRNA H19. *THERANOSTICS* 2018; **8**(14)**:** 3932-3948.

15. Wang J, Xie S, Yang J, Xiong H, Jia Y & Zhou Y *et al.*. The long noncoding RNA H19 promotes tamoxifen resistance in breast cancer via autophagy. *J HEMATOL ONCOL* 2019; **12**(1)**:** 81.

16. Zhang L, Cheng H, Yue Y, Li S, Zhang D & He R. H19 knockdown suppresses proliferation and induces apoptosis by regulating miR-148b/WNT/β-catenin in ox-LDL -stimulated vascular smooth muscle cells. *J BIOMED SCI* 2018; **25**(1)**:** 11.

17. Xiao T, Zou Z, Xue J, Syed BM, Sun J & Dai X *et al.*. LncRNA H19-mediated M2 polarization of macrophages promotes myofibroblast differentiation in pulmonary fibrosis induced by arsenic exposure. *ENVIRON POLLUT* 2021; **268**(Pt A)**:** 115810.

18. Hu XT, Xing W, Zhao RS, Tan Y, Wu XF & Ao LQ *et al.*. HDAC2 inhibits EMT-mediated cancer metastasis by downregulating the long noncoding RNA H19 in colorectal cancer. *J Exp Clin Cancer Res* 2020; **39**(1)**:** 270.

19. Singh N, Ramnarine VR, Song JH, Pandey R, Padi S & Nouri M *et al.*. The long noncoding RNA H19 regulates tumor plasticity in neuroendocrine prostate cancer. *NAT COMMUN* 2021; **12**(1)**:** 7349.

20. Wu X, Sui Z, Zhang H, Wang Y & Yu Z. Integrated Analysis of lncRNA-Mediated ceRNA Network in Lung Adenocarcinoma. *FRONT ONCOL* 2020; **10:** 554759.

21. Wang L & Qi L. The role and mechanism of long non-coding RNA H19 in stem cell osteogenic differentiation. *MOL MED* 2021; **27**(1)**:** 86.

22. Beermann J, Piccoli MT, Viereck J & Thum T. Non-coding RNAs in Development and Disease: Background, Mechanisms, and Therapeutic Approaches. *PHYSIOL REV* 2016; **96**(4)**:** 1297-1325.

23. Yu C, Li L, Xie F, Guo S, Liu F & Dong N *et al.*. LncRNA TUG1 sponges miR-204-5p to promote osteoblast differentiation through upregulating Runx2 in aortic valve calcification. *CARDIOVASC RES* 2018; **114**(1)**:** 168-179.

24. Shi S, Song L, Yu H, Feng S, He J & Liu Y *et al.*. Knockdown of LncRNA-H19 Ameliorates Kidney Fibrosis in Diabetic Mice by Suppressing miR-29a-Mediated EndMT. *FRONT PHARMACOL* 2020; **11:** 586895.

25. Lv M, Zhong Z, Huang M, Tian Q, Jiang R & Chen J. lncRNA H19 regulates epithelial-mesenchymal transition and metastasis of bladder cancer by miR-29b-3p as competing endogenous RNA. *Biochim Biophys Acta Mol Cell Res* 2017; **1864**(10)**:** 1887-1899.

26. Tang H, Zhong H, Liu W, Wang Y, Wang Y & Wang L *et al.*. Melatonin Alleviates Hyperglycemia-Induced Cardiomyocyte Apoptosis via Regulation of Long Non-Coding RNA H19/miR-29c/MAPK Axis in Diabetic Cardiomyopathy. *Pharmaceuticals (Basel)* 2022; **15**(7).

27. Sun B, Ding Y, Jin X, Xu S & Zhang H. Long non-coding RNA H19 promotes corneal neovascularization by targeting microRNA-29c. *Biosci Rep* 2019; **39**(5).

28. Huang X, Wang Z, Li D, Huang Z, Dong X & Li C *et al.*. Study of microRNAs targeted Dvl2 on the osteoblasts differentiation of rat BMSCs in hyperlipidemia environment. *J CELL PHYSIOL* 2018; **233**(9)**:** 6758-6766.

29. Hrdlicka HC, Pereira RC, Shin B, Yee SP, Deymier AC & Lee SK *et al.*. Inhibition of miR-29-3p isoforms via tough decoy suppresses osteoblast function in homeostasis but promotes intermittent parathyroid hormone-induced bone anabolism. *BONE* 2021; **143:** 115779.

30. Liu Y & Zhou Y. Circ_0087960 stabilizes KDM5B by reducing SKP2 mediated ubiquitination degradation and promotes osteogenic differentiation in periodontal ligament stem cells. *REGEN THER* 2022; **19:** 122-130.

31. Kim JW, Simmer JP, Lin BP, Seymen F, Bartlett JD & Hu JC. Mutational analysis of candidate genes in 24 amelogenesis imperfecta families. *EUR J ORAL SCI* 2006; **114 Suppl 1:** 3-12, 39-41, 379.

32. Zhao Z, Han Y, Zhang Z, Li W, Ji X & Liu X *et al.*. Total glucosides of paeony improves the immunomodulatory capacity of MSCs partially via the miR-124/STAT3 pathway in oral lichen planus. *BIOMED PHARMACOTHER* 2018; **105:** 151-158.

33. Cao Y, Xu J, Wen J, Ma X, Liu F & Li Y *et al.*. Generation of a Urine-Derived Ips Cell Line from a Patient with a Ventricular Septal Defect and Heart Failure and the Robust Differentiation of These Cells to Cardiomyocytes via Small Molecules. *CELL PHYSIOL BIOCHEM* 2018; **50**(2)**:** 538-551.

34. Draper JM & Vivian JL. Reprogramming of Primary Human Cells to Induced Pluripotent Stem Cells Using Sendai Virus. *Methods Mol Biol* 2020; **2066:** 217-234.

35. Garg S, Dutta R, Malakar D, Jena MK, Kumar D & Sahu S *et al.*. Cardiomyocytes rhythmically beating generated from goat embryonic stem cell. *THERIOGENOLOGY* 2012; **77**(5)**:** 829-839.

36. Jiang YF, Chen M, Zhang NN, Yang HJ, Rui Q & Zhou YF. In vitro and in vivo differentiation of induced pluripotent stem cells generated from urine-derived cells into cardiomyocytes. *BIOL OPEN* 2018; **7**(1).

37. Kajahn J, Gorjup E, Tiede S, von Briesen H, Paus R & Kruse C *et al.*. Skin-derived human adult stem cells surprisingly share many features with human pancreatic stem cells. *EUR J CELL BIOL* 2008; **87**(1)**:** 39-46.

38. Gnedeva K, Vorotelyak E, Cimadamore F, Cattarossi G, Giusto E & Terskikh VV *et al.*. Derivation of hair-inducing cell from human pluripotent stem cells. *PLOS ONE* 2015; **10**(1)**:** e116892.

39. Lieberman R, Levine ES, Kranzler HR, Abreu C & Covault J. Pilot study of iPS-derived neural cells to examine biologic effects of alcohol on human neurons in vitro. *ALCOHOL CLIN EXP RES* 2012; **36**(10)**:** 1678-1687.

40. Xhabija B & Kidder BL. KDM5B is a master regulator of the H3K4-methylome in stem cells, development and cancer. *SEMIN CANCER BIOL* 2019; **57:** 79-85.

41. Zhang SM, Cai WL, Liu X, Thakral D, Luo J & Chan LH *et al.*. KDM5B promotes immune evasion by recruiting SETDB1 to silence retroelements. *NATURE* 2021; **598**(7882)**:** 682-687.

42. Cao Y, Qiu Y, Liu MX, Hu Y & Chen FW. MiR-29c-3p reduces bone loss in rats with diabetic osteoporosis via targeted regulation of Dvl2 expression. *Eur Rev Med Pharmacol Sci* 2021; **25**(2)**:** 636-642.

43. Martinez I, Cazalla D, Almstead LL, Steitz JA & DiMaio D. miR-29 and miR-30 regulate B-Myb expression during cellular senescence. *Proc Natl Acad Sci U S A* 2011; **108**(2)**:** 522-527.

44. Shang J, Yao Y, Fan X, Shangguan L, Li J & Liu H *et al.*. miR-29c-3p promotes senescence of human mesenchymal stem cells by targeting CNOT6 through p53-p21 and p16-pRB pathways. *Biochim Biophys Acta* 2016; **1863**(4)**:** 520-532.
